# Pencil cleaning technique for robotic liver parenchymal transection: a step further to systematization beyond the microfracture-coagulation method

**DOI:** 10.1007/s11701-025-02480-5

**Published:** 2025-07-17

**Authors:** Jordi Navinés-López, Fernando Pardo Aranda, Manel Cremades Pérez, Alba Zárate Pinedo, Sara Sentí Farrarons, Victoria Lucas Guerrero, Francisco Espin Álvarez, Esteban Cugat Andorrà

**Affiliations:** Autonomous University of Barcelona, Hospital Universitari Germans Trias i Pujol de Badalona, Barcelona, Spain

**Keywords:** Liver surgery, Real robotic approach, Robotic liver surgery, Pedicle driven approach, Liver parenchymal transection, Microfracture-coagulation, Pencil cleaning

## Abstract

Robotic liver resections based on Laennec’s capsule pedicular driven dissection is a challenging surgical procedure, because of the intrinsic relationship between the liver parenchyma and the main intrahepatic vessels. The absence of anatomical landmarks and the frailty of the fine vascular structures requires broad experience, anatomical knowledge and refined technical skills to successfully perform such a procedure. As robotic platforms do no offer the standard laparoscopic advanced tools for liver resection, real robotic approach has been proposed as a default technique for the liver parenchymal transection, precise robotic anatomical and parenchymal-sparing liver resection, and pedicle driven dissection, like the microfracture-coagulation method. A more specific refinement of the real robotic approach, similar to the pencil cleaning process, useful in very careful deep dissections, is defined towards the standardization of the real robotic approach in advanced liver surgery. The rationale of the method, its indications and tips and tricks are described in detail.

**Supplementary Information:**

The online version contains supplementary material available at 10.1007/s11701-025-02480-5.

## Introduction

Robotic liver resection (RLR) is gaining fast worldwide implementation as minimally invasive liver surgery over the laparoscopic approach, although being considered a “non-inferior approach” and a “development in progress” technique [1]. Notwithstanding, despite the lack of high-quality evidence, the implementation of robotic platform advantages push experienced surgeons to perform increasingly complex and demanding hepatic procedures [2].

Nonetheless, liver parenchymal transection (LPT) is still one of the most technically demanding and challenging steps during advanced RLR. The lack of external nor internal anatomical landmarks, homogeneous methodology, and the heterogeneous use of different transection tools [3], leads to an important bias that makes it difficult to compare clinical outcomes between different surgical centers [4], but also to achieve a consensus in its standardization.

According to the available literature, RLR can be classified as hybrid or robotic-assisted, totally or fully robotic, and real or pure robotic.

On one hand, hybrid or robotic-assisted is considered “two-surgeon technique”, because the scrubbed assistant surgeon plays a leading role with laparoscopic tools through the accessory assistant trocar, usually the CUSA dissector. This method may be limited due to its zero degrees of wrist movement, representing a challenging step for the scrubbed surgeon, because of the arch restrictions from the accessory trocar, but leaving apart the full potential of dissection of the robotic arms.

On the other hand, totally or fully robotic approaches are considered “one surgeon techniques”, because the leading surgeon performs the whole procedure from the console, with no use of external laparoscopic tools. The limitation in that case is not to count on the advanced laparoscopic tools, nor the CUSA.

While the totally or fully robotic approach includes the use of the full catalog of advanced energy instruments, just as the Synchroseal®, the real robotic (RR) or pure robotic approach, is also a “one surgeon technique”, but only counting on the platform’s standard default tools (mainly forceps and scissors), and the selective use of the bipolar and monopolar energy, respectively, what can be defined as the minimum common technical option available to perform the RR LPT [5]. This technical baseline represents a highly reliable option to set a standardization.

The WHO has defined standardization as the process of “developing, agreeing upon and implementing uniform technical specifications, criteria, methods, processes, designs or practices that can increase compatibility, interoperability, safety, repeatability and quality” [6]. This need goes through audit and mature reflection when introducing a new surgical technique [7]. As such, setting standards to improve quality and reach the next level of care is key to ensure a rollback of the wheel of continuous improvement [8], embracing the IDEAL framework [9].

Assuming that to set a standard RR LPT is the key milestone to improve the comparability of outcomes, as well as facilitate reliable comparisons between hepatobiliary centers worldwide, it is arguable to specify the methodology in terms of safety and reproducibility. This way, the easiest path to reproducibility is to state basal techniques, and to define its steps, to set the minimum common approach from which to implement technical improvements.

## Objectives

To describe in detail a refined technical option for advanced RLR, specially indicated for fine dissection in deep liver resections, developed upon the systematic use of robotic approach for LPT, what we may refer to as pencil cleaning systematic.

Aiming to implement the RR LPT methodology, the procedure for precise anatomical and parenchymal-sparing liver resection, based on Laennec’s capsule (LC) and Glisson’s sheath (GS) pedicular driven dissection, is reviewed in detail to state the key points linked to standardization.

## Anatomical basis of the robotic liver parenchymal resection

The modern anatomical liver parenchymal division described by Couinaud [10] do not offer visible hands-on limits between each segment on the liver surface, nor into the parenchyma, making the resection challenging due to the high risk of vascular injury. The plate system confirmed the LC limits between the main hepatic veins and the vascular adventitious layer, proving it has no continuity with the GS, but extending to the peripheral pedicles from the Glisson’s limiting hepatic hilar plate. This point was later confirmed in histopathological studies [11], stating the basis for the pedicle driven approach (PDA), which defines up to 6 access gates to initiate the parenchymal transection [12].

Notwithstanding, anatomical variations may increase the complexity of the proper identification of the anatomical resection planes during resection. Hjörtsjo [13] introduced the concept of ventro-dorsal segmentation, defining the vertical fissure, and Takasaki [14] the anatomical conception of the “broccoli” model of the liver, scaled up this approach adding 6 to 8 cone units per segment, pushing forward the parenchyma sparing liver resections feasibility.

These anatomical insights, are especially useful in caudal approach [15, 16], are the rationale basis of the PDA, through caudal approach using the pedicular gates, for isolating transparenchymally the Glissonean pedicles (GP) and the main hepatic veins under Pringle maneuver clamping, but also to mobilize the liver, and to perform the piggy-back and Hanging maneuvers.

In order to optimize the success using PDA, it is highly recommended to improve the surgical planning using high quality imaging and implement it with 3D modeling [17, 18], as a way of performing navigation assisted transection, handling the complexity of the subsegmental anatomy.

As such, the eventual success during the LPT depends upon the proper combination of the intraoperative anatomical insights, the exhaustive preoperative planning, and a refined systematic technique, that, although having no defined approach for the transparenchymal dissection, it has become the key step to standardize the minimally invasive approach.

## Fundamentals of the real robotic approach for the liver parenchymal transection

Assuming optimal patient selection, preoperative prehabilitation, surgical planning and equipment, the RR LPT, based on the use of only basic robotic instruments, proceeds with the selective use of the bipolar and monopolar energy. Monopolar Scissors powered by EndoWrist® (Intuitive) technology allows manipulation in 570°, increasing the articulation and precision, making the energized scissors extremely effective for dissection and hemostasis in complex dissection. Bipolar grasper (Maryland or Fenestrated forceps) also utilizes the EndoWrist® technology, being a versatile instrument for grasping and retracting tissue that doubles as a coating device to achieve hemostasis of small vessels [19].

After Pringle maneuver preparation (with or without selective clamping), the GS is incised, making a 1–2 cm fence along the transection line with the curved monopolar scissors. The navigation tools are checked in-console (i.e., intraoperative ultrasound, ICG dye staining, or 3D model consultation).

RR approach exposes the main GP with bipolar or Maryland forceps to surround it 360º with a vessel loop before transect it with the robotic Endowrist stapler. The second and third GP are taken after locked polymer clips before section. On the other hand, the RR approach for the isolation of the main hepatic veins with following the LS is challenging due to the increased risk of hemorrhage. Using the LC plane, the main trunks of the hepatic veins can be exposed and pursued to their roots. Whether following the main hepatic veins or cutting through the parenchyma, it is possible to access the liver fine vital structures only with default robotics tools.

The former crush-clamp technique was rapidly replaced by more refined techniques, to preserve the hemostasis during the LPT. Very early reports defined the minimalist and most intuitive of these techniques, like the “scissor hepatectomy" [20], two bipolar transection [21] or the “microfracture-coagulation” method (MFC) [5], establishing that 3 to 5 mm vessels, can be taken with monopolar or bipolar energy, and larger vessels can be clipped and/or ligated. The vessels up to 15 mm may be isolated by cold dissection in a segment wide enough to apply medium-large locked clips with the robotic applier, while first and second order GP may be identified, dissected, surrounded with a loop, and lifted up, before placing the wristed robotic endostapler (SureForm wristed da Vinci blue reload 45–60 mm staplers). Main hepatic veins root dissection may be transparenchymal during major hepatectomies, and transected with curved-tip 30 white reload endostapler.

MFC allows transparenchymal progression under PDA, being potentially used in both anatomical and parenchyma-preserving resections. MFC differs essentially from the crush-clamp technique in that the parenchyma is not crushed at all, but fractured in very small cold steps towards deep, so structures can be revealed carefully without being injured. As formerly described, MFC systematic may be subdivided into three consecutive steps: first step cold progression, where the separation of the tooltips fractures the parenchyma, second step bipolar energy selective diathermy for vessels less than 5 mm, and third step monopolar energy, as the curved scissors coagulates the new transection frontline before proceeding to repeat the series.

After performing the initial incisional fence along the superficial limit of resection, the dissection enters a deeper plain, that may be sagittal or coronal, depending on the oncological margin. Dissection of 1st and 2nd order biliary and hepatic branches leads to an extremely challenging need for bloodless exposure, leading to a more refined systematic, that reminds the pencil cleaning.

## Pencil cleaning systematic

Pencil cleaning systematic (PCS) dissection technique is a one-surgeon refined systematic RR approach for LPT and PDA, designed to dissect with maximum precision the vital structures in deep surgical liver dissection. The PCS development arises from the systematic use of the RR approach in liver surgery. RR approach was initially standardized as MFC, described elsewhere, and refined during the latter period of the RR LPT series. This systematic, evolved from the former MFC technique, outperforms its intrinsic limitations, such as the need for iterative check of the transection plane, and the critical view of very small structures in deep liver dissection (see video sample).

PCS not only improve the precision, but also maximize the exposure of the resection margin, avoiding the concave liver parenchymal penetration that may be disadvantageous for incidental bleeding, maintaining the convex exposure of the transection frontline, to keep the vascular control at all times, close similar to the laparoscopic approach, but using the monopolar scissor as a pencil. The move with the monopolar scissors is performed mainly cold (with no energy), to clean up the smallest of detail and remove the coagulated surface layer to reveal the smaller hidden vessels, in an iterative gentle scissor move. Monopolar energy may be applied very selectively, mainly to assist the dissection rather than apply hemostatic coagulation.

**Fig.1:**
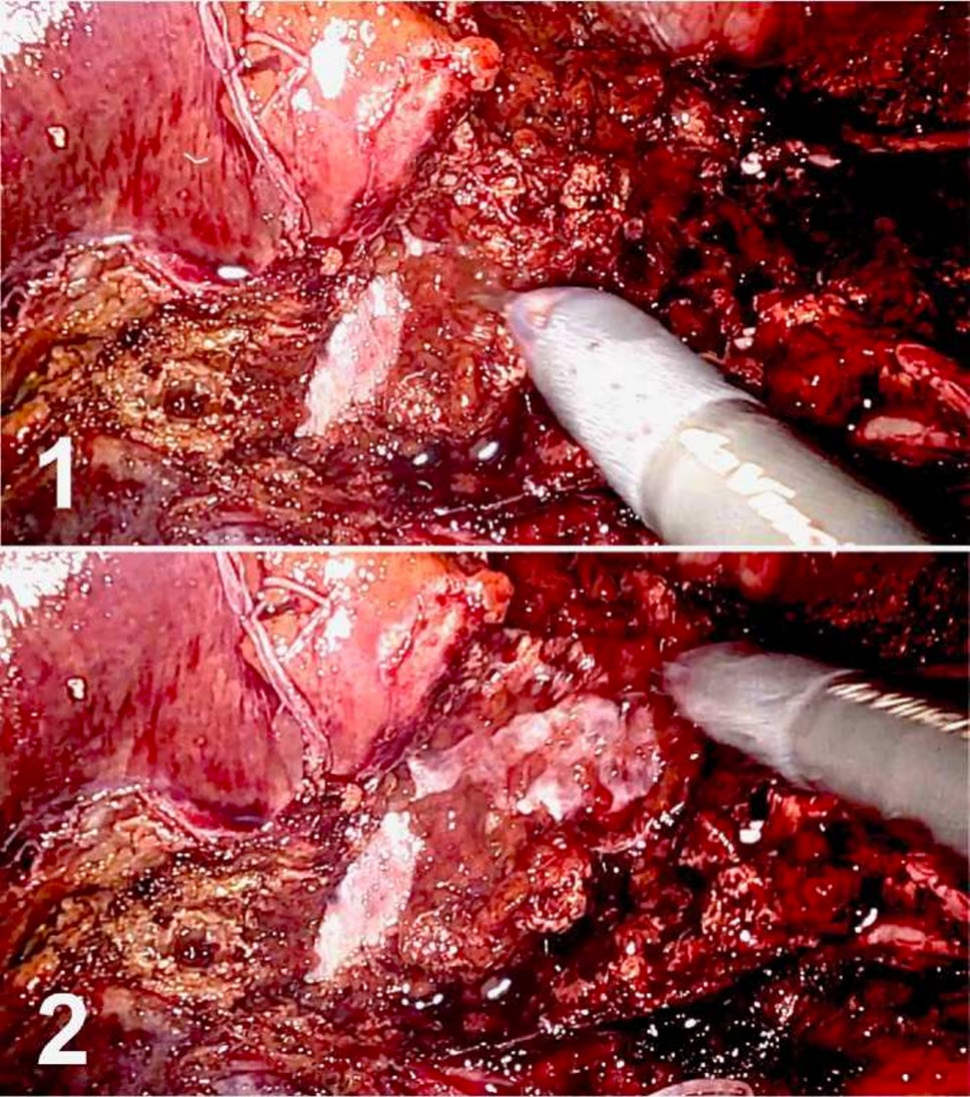
Pencil cleaning systematic dissection technique. 1: closed scissor in contact with the main middle vein, 2: closed scissor brushes the vessel wall like a pencil, uncovering the main middle vein

The move resembles the pencil cleaning of the fine work, gently using the plugged closed scissor as a soft brush or a *clean*, to remove softly the spurious friable liver tissue before small fragile collaterals. This way, tool tip brushes out from deep to outer layers, avoiding making deep holes during the dissection, but discarding all the minor remains, keeping the vascular control and preserving the small Glissonean pedicles and vascular collaterals (see Fig. 1).

This systematic dissection preserves even the smallest hidden vessels, improving the dissection control over all vascular structures in all transection planes during the LPT, giving the surgeon the option to take it whenever direction may need, after safe bipolar coagulation or clipping.

The final transection surface is also checked at the end of the procedure, just similar to that after MFC. This revision is usually done after releasing the hilar clamp, by applying gauze onto the transection surface, and then removing it rolling over, uncovering one by one the potential oozing points, so superficial bipolar coagulation can be applied selectively, avoiding monopolar coagulation that could leave ischemic bedsores areas below, and eventually be the origin of potential bilomas or hematomas.

## Results

The series (Table 1) includes 194 robotic liver resections performed in 180 patients for 238 lesions with the Da Vinci Xi Surgical System in all cases, consecutively collected between April 2018 and January 2025. The patients were aged 64.3 (20–85) years (113 men, 67 women) BMI 29.8 (17.1–41.4), ASA 2.4 (44.2% > 3) and Charlson comorbidity index 6.7 (1–13).

**Table 1:** Sample series. Baseline descriptive and perioperative data

Descriptive data	RLS (*n* = 180)
Preoperative baseline characteristics	
Age, year, median (IQR)	64.3 (20–85)
Female, *n*º (%)	67 (37.2)
BMI, kg/m2, median (IQR)	29.8 (17.1–41.4)
ASA, *n*º (%) I–II	101 (56.1)
ASA, *n*º (%) III–IV	79 (43.9)
Charlson CI, median (IQR)	6.7 (1–13)
Etiology	
Malignant, *n*º (%)	158 (87.8)
CRCM	89
NCRCM	10
HCC	38
IHCC	13
EHCC	1
GBC	7
Adenoma	5
Cyst, complex	10
Miscellaneous (other)	17
Intraoperative	
Resections, *n*º	194
Lesions, *n*º	238
Lesions in posterior segments (6,7,8), *n*º (%)	83 (46.1)
Size in mm, median (IQR)	28.1 (4–85)
Major liver resections, *n*º (%)	20 (11.1)
Right hemihepatectomy, *n*º (%)	9 (5.0)
Left hemihepatectomy, *n*º (%)	9 (5.0)
Central hepatectomy, *n*º (%)	2 (1.1)
Anatomic minor liver resections, *n*º (%)	102 (56.7)
Left lateral sectorectomy, *n*º (%)	41 (22.8)
Bisegmentectomy (other), *n*º (%)	7 (38.9)
Segmentectomy, *n*º (%)	45 (25.0)
Sectorectomy (other), *n*º (%)	9 (5.0)
Parenchyma-sparing liver resections, *n*º (%)	57 (31.7)
Operative time, median (IQR)	220.5 (32–510)
Pringle hilar clamping time, median (IQR)	53.2 (2–123)
Conversions, *n*º (%)	8 (4.4)
Transfusions, *n*º (%)	9 (5.0%)
Blood loss (ml), median (IQR)	161.0 (50–900)
R0 oncological free margin, *n*º (%)	138 (86.3)
Margin (mm), median (IQR)	9.1 (1–54)
Postoperative	
Length of hospital stay in days, median (IQR)	4.7 (2–58)
Reintervention, *n*º (%)	3 (1.7)
Severe morbidity (Clavien-Dindo ≥ 3), *n*º (%)	13 (7.2)
ISGLS Bile leakage grade B/C, *n*º (%)	3 (1.7)
Mortality < 90 days postoperative, *n*º (%)	3 (1.7)

*RLS* robotic liver surgery, *ASA* American society of anesthesiologists physical status classification system score, *BMI* body mass index, *CCI* Charlson comorbidity index, *CRCM* colorectal cancer metastases, *NCRCM* non colorectal cancer metastases, *HCC* hepatocellular carcinoma, *IHCC* intra-hepatic cholangiocarcinoma, *EHCC* extra-hepatic cholangiocarcinoma, *GBC* gallbladder cancer, *ISGLS* international study group of liver surgery

Indication for malignancy was in 158 (87.8%) cases (89 colorectal metastases, 38 hepatocellular carcinoma, 13 intrahepatic cholangiocarcinoma, 10 non-colorectal metastases, 8 gallbladder cancer, and 1 Klatskin tumor).

Surgical resections were predominantly (69%) anatomical (45 anatomical segmentectomies, 41 left lateral sectorectomies, 7 bisegmentectomies, 6 posterior sectorectomies and 9 anterior sectorectomies), 20 major hepatectomies, (2 central, 9 left, 7 right hemihepatectomies, 2 ALPPS), and 57 limited parenchyma resections.

**Fig. 2:**
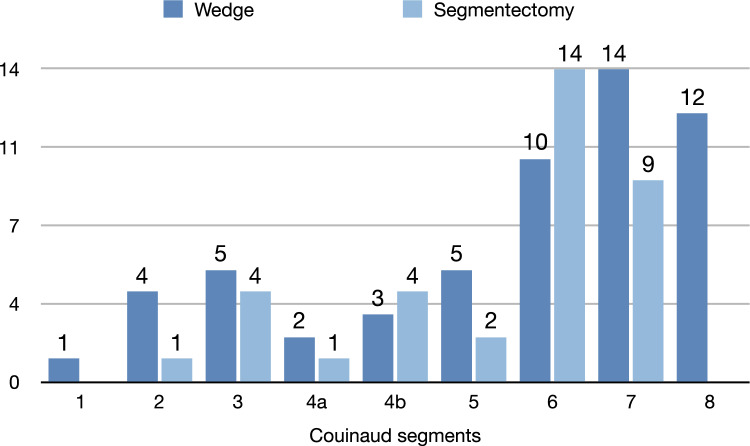
Minor anatomic (segmentectomy) and parenchyma-sparing (wedge) cumulative robotic liver resections histogram

There were 109 resected lesions in posterior segments 6, 7, 8 (45.8%), but 134 in the right lobe (56.3%). The combined distribution of wedge resections and anatomical segment resections show a clear predominance (66.7%) of resections in the posterior segments (6–7-8), considered difficult by laparoscopic approach (See Fig. 2).

Operative time was median 220.5 min, with a Pringle time of median 53.2 min, used in the 95% of cases. Mean blood loss was median 161 ml. Nine patients received perioperative transfusion, median 2.4 units (1–5). The total hospital stay was median 4.7 days. Severe Clavien–Dindo 90-day morbidity was observed in 13 cases (7.2%), median Comprehensive Complication Index 22.6, with 14 cases of postoperative liver failure (ISGLS 7A, 3B, 3C), 4 bile leaks (ISGLS 1A, 3B), 4 hemorrhage (ISGLS 1A, 2B, 1C), 3 surgical site infections.

The eight cases of conversion were due to: one to laparoscopy (energy failure), and seven to open surgery (three adhesion syndrome, two bleeding control, one Pringle intolerance, one diaphragmatic resection). There were three cases of re-intervention (1 laparoscopic intestinal deworming prior to docking, 1 intestinal obstruction, 1 hemoperitoneum after ALPPS) and 3 cases of mortality (ISGLS grade 3 postoperative liver failure after resection).

## Discussion

MFC and PCS both are cold RR blunt techniques that differ essentially from crush-clamp technique, as they do not crush the liver parenchyma, but splitting (in the case of MFC) or cleaning (in the case of PCS). This results in a preserving dissection systematic that optimizes the possibilities to identify small intraparenchymal vessels.

Although it can be assumed that the pencil cleaning movement itself may be intuitively performed by many surgeons, the authors’ objective is to depict the key specific features of what is meant to be a real robotic liver transection, to take a definition from it, in order to systematize the RR approach towards the standardization.

PCS is characterized by the dissection move, not splitting but cleaning up the parenchyma just before fine structures, identifying but preserving it at the same time. It is specially indicated in assessing the dissection plane direction and position before applying iteratively MFC in case of deep dissection into the parenchyma, and also when vital structures need to be carefully dissected, just as the main hepatic veins.

PCS also has the exclusive feature of following the dissection plane according to the Laennec’s PDA 22, thus avoiding the need for iterative check of the resection margin, but also preserving the deliberate desirable oncological free margin during all steps of the dissection. It allows to progress through the surgical desired plane, regardless of the parenchymal fracture plane, which in case of MFC needs to be reassessed when approaching a malignant lesion, due to its intrinsic dependency of the direction taken by the stromal architectural collagen fibers during the LPT. PCS can progress completely independent from the effect produced by the occult lesion over the adjacent liver parenchyma, thus implementing the security of the oncological free margin.

The parenchymal transection progress is made of small cold steps in all phases along the liver resection procedures, so to achieve this under bloodless conditions it is mandatory to maintain a very low CVP during all the procedure, and a close collaboration with the anesthesiologist team is core to fulfill it successfully. Furthermore, the avoidance of crush-clamping allows the surgeon to properly identify all significant vessels with no injury, obtaining a bloodless surgical field that helps the dissection, thanks to the absence of blood spillage. As PCS does not crush the parenchyma, it preserves both LS and GC, to achieve a high grade of fine vascular dissection and control, thus optimizing the bloodless surgical field, what is the reason for an even more bloodless final transection field, which may result in a decrease of the final hemostasis check operative time and blood loss. Saline irrigation is not required before bipolar forceps cauterization during de LPT, nor monopolar energy cauterization, but after releasing the hilar clamp saline may be used to clean up the transection surface, which may help to maximize the bipolar coagulation efficiency during the hemostasis coverage, previous to leaving the procedure ended.

As PCS do not substitute MCF as a method of LPT, is not possible to generate spliced data suitable for comparison between the two methods. The authors consider that both methods are distinguishable, but complementary as repeatable and systematized, thus simultaneously applicable to any advanced RLR. Although there are many options for it, the RR liver parenchymal transection obtains a bloodless resection surface comparable to that obtained by laparoscopic approach. The refinement of the transparenchymal cold dissection is a major step towards standardization. Its systematization opens the possibility of considering the RR techniques as a baseline default transection technique option to perform the LPT in advanced RLR, what may eventually result in comparable outcomes, thus unlocking the possibility of high quality healthcare reviews on the topic.

## Conclusions

PCS can be defined as a safe, reproducible and compatible technique, different from MFC, especially indicated for deep dissection of LPT and fine fragile vital structures with RR approach. This characteristics make the RR approach for LPT suitable for systematization.

It is the authors’ recommendation to promote the RR techniques as the default standard baseline option to perform the LPT in advanced RLR.

## Supplementary Information

Below is the link to the electronic supplementary material.

Supplementary file1 (MP4 199313 kb)

## Data Availability

No datasets were generated or analysed during the current study.
